# NanoArduSiPM: A Miniaturized Integrated Platform for Scalable Scintillation-Based Particle Detection

**DOI:** 10.3390/s26103135

**Published:** 2026-05-15

**Authors:** Valerio Bocci, Giacomo Chiodi, Francesco Iacoangeli, Alberto Merola, Luigi Recchia, Roberto Ammendola, Davide Badoni, Marco Casolino, Laura Marcelli, Gianmaria Rebustini, Enzo Reali, Matteo Salvato

**Affiliations:** 1 INFN Sezione di Roma, Dipartimento di Fisica, Università “La Sapienza”, Piazzale Aldo Moro 2, 00185 Rome, Italy; giacomo.chiodi@roma1.infn.it (G.C.); francesco.iacoangeli@roma1.infn.it (F.I.); alberto.merola0@gmail.com (A.M.); luigi.recchia@roma1.infn.it (L.R.); 2 INFN Sezione di Roma “Tor Vergata”, Via Della Ricerca Scientifica 1, 00133 Rome, Italy; roberto.ammendola@roma2.infn.it (R.A.); davide.badoni@roma2.infn.it (D.B.); marco.casolino@roma2.infn.it (M.C.); laura.marcelli@roma2.infn.it (L.M.); gianmaria.rebustini@roma2.infn.it (G.R.); enzo.reali@roma2.infn.it (E.R.); matteo.salvato@roma2.infn.it (M.S.)

**Keywords:** silicon photomultiplier, SiPM, CubeSat instrumentation, scintillator, ArduSiPM, SoC, microcontroller, IoT, space

## Abstract

NanoArduSiPM represents a paradigm shift in the ArduSiPM (Architected Detection Unit for Silicon Photomultipliers) roadmap, evolving from a standalone instrument into a high-density modular building block (36 mm × 42 mm × 3 mm, 7 g). This revision does not merely pursue miniaturization; it re-engineers the signal-processing chain to maintain high performance within a scaled-down footprint, enabling the transition from single-unit detection to scalable, distributed multi-detector systems. NanoArduSiPM is based on a three-layer architecture comprising an external scintillator and Silicon Photomultiplier (SiPM) detection module, a dedicated high-speed discrete analog front-end, and a System-on-Chip (SoC) for embedded acquisition and processing. The physical implementation adopts high-integrity PCB routing and rigorous isolation techniques designed to suppress digital–analog coupling, a critical requirement in such a compact form factor. This deterministic layout strategy provides the architectural foundation for time-tagging capabilities, currently under quantitative characterization, by addressing the fundamental sources of signal interference at the hardware level. Beyond hardware integration, NanoArduSiPM introduces the capability for extended firmware functionality, including event tagging via external inputs and the implementation of coincidence and veto logic. This framework supports the acquisition of multiple correlated histograms and allows multiple units to be interconnected on a shared SPI bus. By shifting from standalone operation to a coordinated, hierarchical architecture, NanoArduSiPM enables distributed detection schemes where event selection and correlation are handled natively within the system, reducing the dependency on external data acquisition electronics. The compact modular architecture, together with the high-performance discrete analog front-end and embedded data handling, makes NanoArduSiPM suitable for applications where low mass and low power consumption are critical, targeting applications such as space-based payloads, laboratory instrumentation, remote sensing, and large-scale distributed multi-channel detection systems. While no radiation-tolerance qualification of the complete system has been performed in this work, the microcontroller family used in the design is also available in radiation-tolerant variants, which may support future implementations targeting more demanding radiation environments.

## 1. Introduction

Over the last decade, advances in Silicon Photomultipliers (SiPMs) [1] and mixed-signal microcontrollers have enabled increasingly compact scintillation-based detectors with embedded data processing capabilities [2,3,4]. These developments have progressively shifted radiation and particle detection systems from centralized data acquisition architectures toward highly integrated instruments, suitable for deployment in space platforms, distributed monitoring networks, and resource-constrained environments.

Such compact and self-contained detection systems are increasingly adopted in space-based and CubeSat radiation monitoring instrumentation, as demonstrated by missions such as RADMON and CELESTA [5,6].

Following these developments, and the introduction of the ArduSiPM concept [2,3,4] in the early 2010s, several compact particle and radiation detection systems have been proposed in the literature [7,8,9,10,11,12,13]. Since its first implementation, ArduSiPM has evolved across multiple hardware generations while preserving its core architectural principle: exploiting the internal peripherals of modern System-on-Chip (SoC) to implement complete detection and data processing chains without the need for external acquisition units. The ArduSiPM architecture has already been successfully deployed in a broad range of real-world applications, including distributed radiation monitoring and laboratory-scale scintillation measurements. Its versatility has been demonstrated in low-light photonic applications such as bio- and chemiluminescence analysis [14,15], as well as in biomedical radiological instrumentation in clinical environments [16].

NanoArduSiPM represents the third-generation evolution of the ArduSiPM technology [2,3,4]. Compared to the previous CosmoArduSiPM implementation, it introduces a substantial reduction in size and mass, achieving approximately a factor-of-two reduction in size and a threefold decrease in mass (the previous implementation measured 96 × 48 × 3 mm with a mass of 21 g, compared to 42 × 36 × 3 mm and 7 g for the present system), while preserving the all-in-one architectural approach and achieving a measurable and relevant improvement under the constraints imposed by extreme miniaturization in mixed-signal performance. The design goal of this third-generation system was not limited to dimensional scaling, but explicitly targeted the preservation of linearity and count-rate capability, while implementing the architectural framework enabling time tagging. This miniaturization is not merely a mechanical optimization, but a key enabling factor that allows a conceptual transition from a single, self-contained instrument to a compact modular building block. Thanks to its reduced footprint, NanoArduSiPM can be replicated to form scalable detector systems.

Highly integrated single-chip SiPM readout solutions achieve extremely high channel density within very compact footprints, making them particularly effective for applications involving a large number of closely packed channels [17,18,19,20].

In this regime, the NanoArduSiPM architecture is not intended to compete in terms of channel density per unit area.

Instead, it targets a complementary operational domain, where system-level flexibility, distributed operation, and local computational capabilities for event processing and tagging become more relevant than channel density.

By embedding signal processing, digitization, and decision logic within each module, NanoArduSiPM enables distributed multi-detector systems interconnected through digital interfaces, thereby eliminating the need for long analog signal routing and improving robustness against noise and signal degradation.

From a hardware perspective, NanoArduSiPM retains the established ArduSiPM architecture based on a discrete analog front-end coupled to a high-performance SoC, while introducing targeted design refinements driven by miniaturization and signal integrity constraints. In particular, the analog front-end has been redesigned, featuring a low-noise amplifier with an optimized feedback network. This solution provides the flexibility to adapt the system response to different scintillator pulse shapes by modifying only a limited number of passive components, without altering the overall architecture. The detailed circuit implementation and its characterization are discussed in subsequent sections.

Despite the increased integration density and reduced board size, linearity and count-rate performance are preserved, with a discriminator-based architecture supporting synchronization and time-tagging capabilities. These improvements are primarily attributed to enhanced suppression of digital–analog coupling, achieved through optimized PCB routing, grounding strategies, and component placement in a highly constrained mixed-signal environment. Such aspects are particularly critical in SiPM-based detectors, where fast analog signals coexist with high-frequency digital activity on the same board.

At the system level, NanoArduSiPM extends the role of embedded processing beyond conventional data acquisition. The firmware supports event tagging based on external detector signals, as well as coincidence and veto logic that enable synchronized operation of multiple units.

In addition, multiple NanoArduSiPM modules can be connected and read out through a shared SPI bus, allowing compact multi-detector systems with deterministic operation and centralized control, while preserving on-board data processing.

In this context, the tight integration of analog signal conditioning, digitization, and embedded processing makes system-level effects directly relevant for spectroscopic performance. In particular, non-ideal behavior of the internal ADC becomes observable in compact implementations, introducing localized channel inefficiencies under uniform excitation conditions.

This effect can be addressed at the system level through calibration procedures and firmware-based correction, restoring spectral continuity without requiring additional analog circuitry. This demonstrates the role of embedded processing in mitigating intrinsic non-idealities of highly integrated components.

SiPM readout solutions can be divided into (i) high-performance multi-channel ASIC-based systems and (ii) compact single-channel or low-channel-count devices (such as the original ArduSiPM). The former are well suited for systems with high spatial channel density, providing advanced energy resolution and dedicated signal processing, but rely on centralized data acquisition architectures, whereas the latter offer reduced size and complexity with integrated on-board processing.

NanoArduSiPM is a compact single-channel device with fully integrated data acquisition and processing, designed for applications with spatially distributed detection channels. Its architecture enables its use as a building block for complex detector systems, supporting modular configurations, coincidence operation, and distributed topologies, including sparse or large-area detector arrangements.

NanoArduSiPM represents a further evolution of the ArduSiPM concept, with increased integration and enhanced on-board processing capabilities.

While this work primarily focuses on hardware design and characterization, the platform is explicitly conceived as a deployable building block for distributed detection systems, enabling immediate application in multi-node coincidence measurements, compact spectrometry setups, and spaceborne radiation monitoring. The following sections describe the hardware architecture, analog front-end design, firmware features, and experimental characterization that validate this approach. The primary contribution of this work is not the absolute performance of individual components, but the demonstration of a fully integrated, compact, and scalable detection architecture in which analog front-end, digitization, and data processing are co-designed and embedded within a single system.

In this perspective, NanoArduSiPM is presented as a compact system-level solution integrating signal conditioning, digitization, and data processing within a single unit.

The scope of this work is the validation of the system-level architecture and the embedded acquisition and processing chain under miniaturization constraints, rather than an exhaustive experimental benchmarking of all possible configurations.

## 2. The NanoArduSiPM: A Third-Generation Detection Architecture

NanoArduSiPM is the third-generation development of the ArduSiPM technology. It achieves further miniaturization while preserving the all-in-one SoC-based architecture of its predecessors. The system is based on the SAMV71 microcontroller (Microchip Technology Inc., Chandler, AZ, USA) SoC [21], which features a 300 MHz ARM^®^ Cortex^®^-M7 processor in a 9 mm × 9 mm package. Combined with ultra-compact surface-mount components, this results in a highly compact and lightweight board.

**Figure 1:**
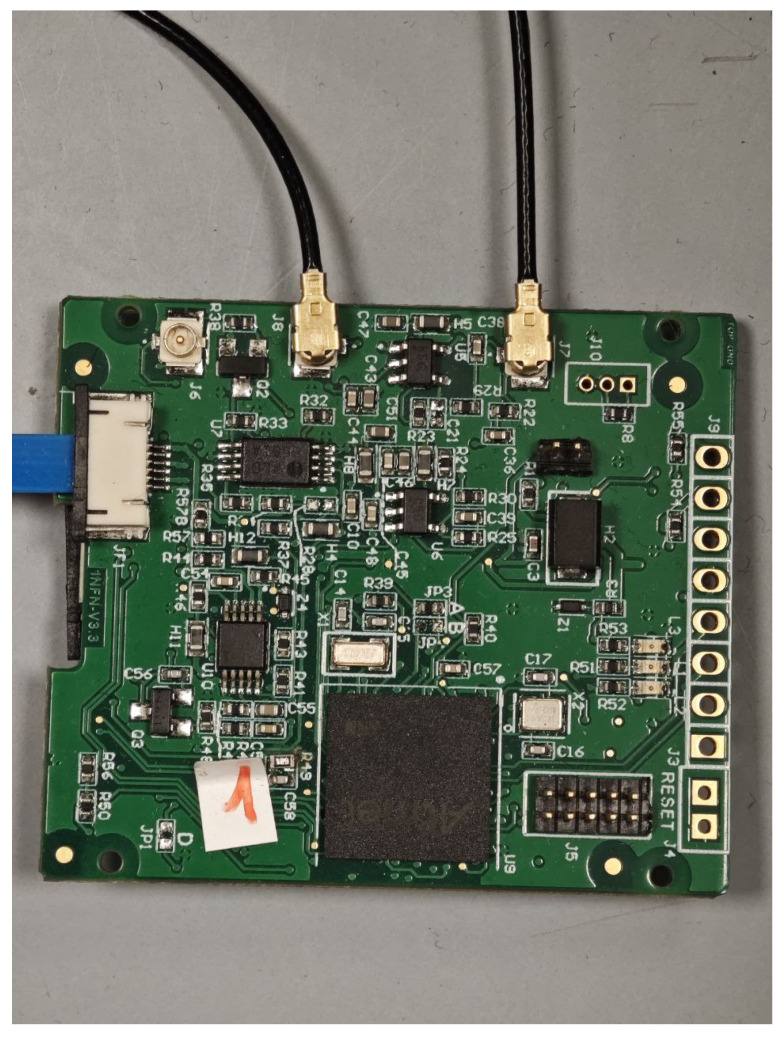
Photograph of the NanoArduSiPM board.

A photograph of the NanoArduSiPM board is shown in Figure 1. The board has been optimized for minimum footprint and mass, yielding dimensions of 42 mm × 36 mm × 3 mm and a total mass of 7 g. A detailed dynamic power consumption analysis is currently ongoing.

Preliminary measurements indicate an average power consumption of approximately 300 mW under continuous acquisition conditions in a static configuration, i.e., without significant event processing load and without exploiting processor standby or low-power optimization features. This represents a conservative estimate, which can be further reduced through firmware-level power management strategies.

The measured consumption varies with firmware configuration and processing load. The total power budget includes contributions from the analog front-end, the high-voltage bias generator, and the SoC processing activity. A detailed characterization under dynamic operating conditions is ongoing. In addition to functioning as a compact standalone detector, NanoArduSiPM is designed as a modular unit for multi-detector systems. The architecture supports the deployment of multiple synchronized units with minimal external infrastructure, enabling distributed and hierarchical detection networks.

The system is intended for embedded applications such as satellite payloads, where all operating voltages are supplied by the platform Electrical Power System (EPS). It includes an onboard high-voltage generator for SiPM biasing, regulated by the processor according to a device-specific calibration curve. A temperature sensor placed near the detector provides automatic bias adjustment to ensure stable operation over varying thermal conditions.

The current implementation is primarily intended for compact space platforms, such as CubeSats and PocketQubes operating in low-Earth orbit, where constraints on mass, volume, and power consumption are dominant and moderate radiation levels can be tolerated. While no radiation-tolerance qualification of the complete system has been performed in this work, the SAMV71 microcontroller family used in the design is also available in radiation-tolerant variants, which may support future implementations targeting more demanding radiation environments.

The signal-processing chain is structured in three layers. The detection layer comprises a scintillator coupled to a Silicon Photomultiplier (SiPM). The SiPM output is amplified by a low-noise analog front-end (LNA) that preserves the nanosecond-scale pulse shape.

The amplified signal is routed to two parallel branches. In one branch, a fast discriminator with programmable threshold (set by the SoC DAC through an analog multiplexer) generates logic pulses that are processed by the timer/counter peripherals of the SAMV71 for event counting and rate measurements.

In the amplitude branch, a peak-hold circuit stretches the SiPM pulse to a microsecond-scale signal suitable for the internal ADC. The digitized amplitude is then analyzed by the embedded CPU. All pulse analysis, histogram generation and data processing are performed entirely within the SoC, eliminating the need for external acquisition electronics.

NanoArduSiPM also supports multi-module configurations via a shared SPI interface, allowing synchronized readout of several units. Four external digital inputs (Tag0–Tag3) enable event tagging, veto and coincidence logic. These signals are handled in real time by the firmware, supporting autonomous event selection and conditional histogramming.

**Figure 2:**
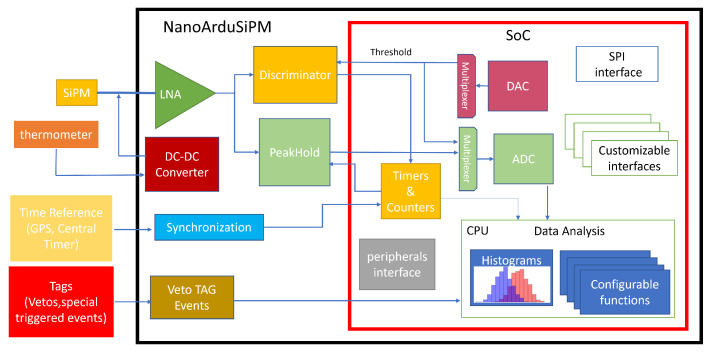
Block diagram of the NanoArduSiPM. Most functions rely on the microcontroller’s internal peripherals, with provision for external reference signals (e.g., GPS or central clock) and logical tags for event selection.

The complete architecture is shown in Figure 2. Signal amplification, discrimination, digitization, event tagging, coincidence handling, histogram generation and processing are all integrated on the same board.

A key integration feature is the use of a Flat Flexible Cable (FFC), which allows flexible positioning of the detector board in compact or stacked assemblies with minimal additional mass and volume. Commercial-grade FFCs with low-outgassing materials and adequate thermal stability have been selected for the planned short-lifetime in-orbit demonstrations.

A dedicated firmware has been developed to support real-time local data processing, distributed synchronization, event selection and onboard data compression, addressing the constraints of bandwidth- and power-limited environments.

In summary, NanoArduSiPM extends the all-in-one SoC-based architecture of previous generations by adding support for scalable distributed operation, embedded event tagging and synchronization capabilities, facilitating coordinated detection networks in space and other resource-constrained applications. The NanoArduSiPM system is designed as a general-purpose, scalable readout platform for SiPM-based scintillation detectors, rather than an application-specific optimized solution. As such, its design is not constrained to a single scintillator type or energy range, but aims to provide flexibility across a broad class of detectors.

From an architectural standpoint, the system has been conceived to support a wider set of features and operating modes than those currently implemented in the firmware. The present work focuses on the validation of the core acquisition chain, while additional functionalities are part of ongoing developments.

The system supports a wide dynamic range compatible with typical SiPM output signals, as confirmed by the linearity measurements presented. The achievable count rate for the ADC-based amplitude measurement is primarily limited by the peak-hold and ADC acquisition chain, resulting in an effective dead time in the range of 2–5 µs. The counter, however, operates independently and is not subject to this limitation.

Bias stability is ensured by the implemented SiPM biasing circuitry, while environmental conditions such as temperature and radiation tolerance are considered application-dependent and are not explicitly addressed in the present implementation.

## 3. Overview of the Discrete Analog Front-End of NanoArduSiPM

This section presents the discrete analog system implemented in NanoArduSiPM, which operates alongside the SoC to ensure signal acquisition, amplification and discrimination. While the overall architecture remains consistent with the original ArduSiPM, the analog stages have been optimized for compactness and compatibility with the reduced form factor of the latest ArduSiPM version.

### 3.1. Low-Noise Voltage Amplifier

When photons are detected, the SiPM generates fast current pulses arising from the avalanche discharge of its microcells, with amplitudes proportional to the number of photoelectrons. The device is reverse-biased, and the resulting current pulse produces a corresponding voltage transient at the readout node defined by the bias and load network. This signal is fed to the analog front-end, whose first stage is a low-noise amplifier (LNA) in inverting configuration, providing low-noise amplification while preserving the nanosecond-scale pulse shape required for amplitude analysis and accurate signal reconstruction.

The SiPM is read out in a cathodic configuration and connected to the acquisition board through a coaxial transmission line. As a result, the signal at the LNA input reflects the combined response of the SiPM, the transmission line, and the input load/bias network. From a circuit perspective, the SiPM current pulse generates a voltage signal across the detector load and bias network, which is then AC-coupled to the front-end through the decoupling capacitor. The operational amplifier operates in inverting configuration, with gain determined by the feedback network $-R_{F}/R_{S}$, where $R_{S}$ represents the effective input resistance seen by the signal. Therefore, the current-to-voltage conversion is distributed within the detector and front-end network rather than occurring at a single localized element, and the signal at the LNA input corresponds to the detector-equivalent voltage after propagation through the readout chain.

The LNA is based on a commercial high-speed operational amplifier (500 MHz bandwidth, low input-referred noise). In inverting configuration, it provides a gain in the range of approximately 5–10, depending on the selected feedback network, while maintaining fast response and low offset. The amplifier feedback network can be adjusted by modifying passive component values, allowing operation either as a direct voltage amplifier for fast signals (e.g., BC408) or as a quasi-integrating stage for slower scintillation signals (e.g., CsI). The feedback network therefore determines both the gain and the frequency response of the stage.

Accordingly, the front-end preserves the integrity of short SiPM pulses in fast-response mode, while allowing controlled integration for slower scintillation processes, enabling the collection of photons arriving at later times due to scintillation decay.

During the calibration phase, the SiPM high-voltage bias is disabled via a dedicated jumper, implemented as a board-level feature, and electrical test pulses are injected at the amplifier input through the same readout connector. These voltage pulses reproduce the equivalent voltage developed at the SiPM readout node after current-to-voltage conversion, enabling direct comparison between controlled test signals and real detector responses.

**Figure 3:**
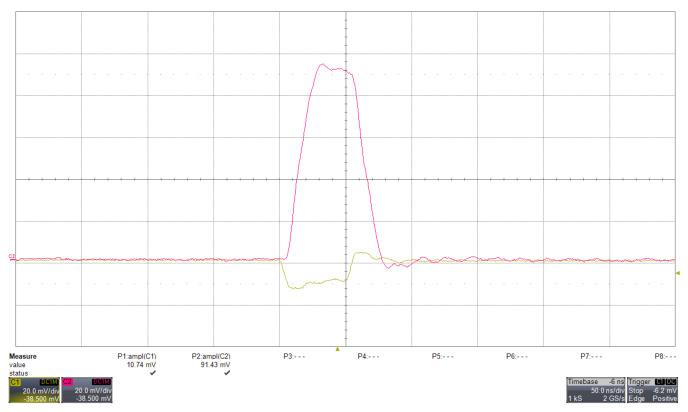
(Colour online) Response of the LNA to a test pulse injected at its input, with the SiPM high voltage disabled. The input signal is generated by a pulse generator and fed directly to the LNA input, replacing the SiPM output. The yellow trace represents the input pulse (10.74 mV amplitude, 60 ns width), while the fuchsia trace shows the corresponding amplified signal measured at the LNA analog output (Ana_out), with an amplitude of 91.43 mV. Both traces are displayed with a vertical scale of 20 mV/div and a horizontal scale of 50 ns/div, as measured with a WaveRunner HRO 64 Zi oscilloscope (Teledyne LeCroy, Chestnut Ridge, NY, USA) (1 GHz bandwidth). The resulting amplification corresponds to a gain of approximately 8.5, illustrating the response of the analog front-end to fast input signals.

The response of the amplifier to a fast input pulse is shown in Figure 3. All waveforms shown are directly measured at the LNA output (Ana_out), which represents the common signal node before branching into the peak-hold and discriminator paths.

During calibration and front-end characterization, the SiPM high voltage is disabled and controlled electrical test pulses are injected at the amplifier input through the detector readout connector. These pulses do not reproduce the microscopic avalanche process inside the SiPM; rather, they reproduce the equivalent voltage transient that would appear at the SiPM readout node after current-to-voltage conversion. This allows the intrinsic response of the analog front-end to be measured under controlled and reproducible conditions, independently of the stochastic fluctuations of real SiPM signals.

### 3.2. Fast Discriminator

A high-speed single-ended comparator is used as a discriminator to detect pulses exceeding a programmable threshold. The selected component features sub-nanosecond time jitter and a propagation delay of approximately 7 ns, making it suitable for high-rate event detection.

The discriminator design was first modeled and optimized through circuit-level simulations, and later refined and validated through laboratory measurements on prototype boards. Achieving the targeted performance required meticulous PCB layout optimization, as the NanoArduSiPM integrates high-speed digital logic tightly coupled with ultra-sensitive analog front-end stages. This mixed-signal environment imposes stringent constraints on grounding topology, signal routing, impedance control, and power distribution, where even minor parasitic effects can significantly degrade signal integrity and noise performance.

The integration of a high-speed 500 MHz front-end amplifier ensures accurate preservation of the pulse leading edge, supporting reliable event triggering and tagging at the system level.

The discriminator-based path is used for event triggering and tagging rather than for precision timing measurements. Consequently, detailed timing effects, such as time walk, have not been quantitatively investigated in this work and will be addressed in future characterization.

The internal counter peripherals of the SAMV71 process the discriminator output. The effective time granularity is defined by the internal clock of the microcontroller and its counter implementation, corresponding to approximately twice the clock period (about 6.7 ns at 300 MHz), due to the internal synchronization and capture mechanism of the counters.

Both the threshold level and pulse width of the discriminator are configurable via firmware.

Furthermore, the discriminator output is buffered and routed to a dedicated U.FL connector, ensuring signal integrity while enabling synchronization with external modules or coincidence logic with additional detection channels.

**Figure 4:**
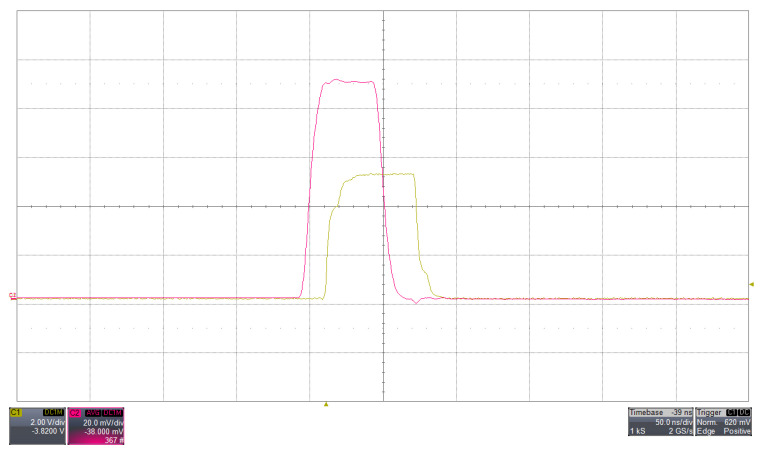
(Colour online) Discriminator response to a test pulse injected at the LNA input with the SiPM high voltage disabled. The fuchsia trace shows the analog signal at the LNA output (Ana_out), while the yellow trace represents the corresponding LVTTL discriminator output. The input pulse has an amplitude of 10.74 mV and a width of 60 ns, with a discriminator threshold set to 8 mV. The analog and digital signals are displayed with vertical scales of 20 mV/div and 2 V/div, respectively, and a horizontal scale of 50 ns/div, as measured with a Lecroy WaveRunner HRO 64 Zi oscilloscope (1 GHz bandwidth).

**Figure 5:**
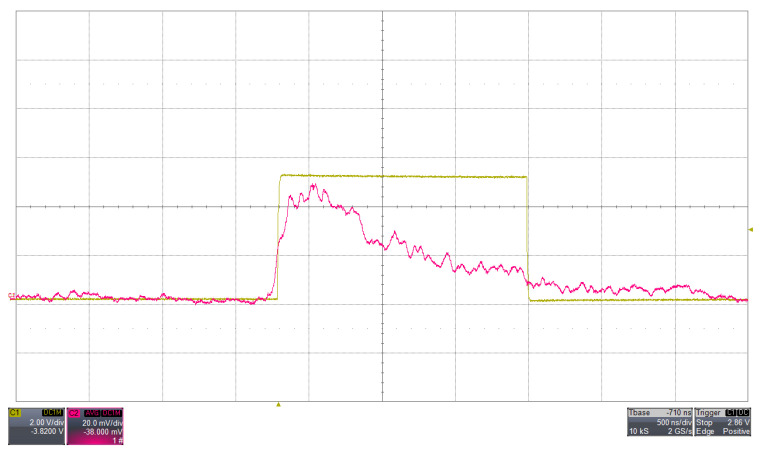
(Colour online) Discriminator response to a real scintillation signal obtained with a CsI(Tl)-coupled SiPM. The fuchsia trace shows the analog signal measured at the LNA output (Ana_out), while the yellow trace represents the corresponding LVTTL discriminator output. The discriminator threshold is set to 8 mV. The signals are displayed with vertical scales of 20 mV/div and 2 V/div for the analog and digital traces, respectively, and a horizontal scale of 500 ns/div, as measured with a Lecroy WaveRunner HRO 64 Zi oscilloscope (1 GHz bandwidth). Compared to the test pulse response, the SiPM signal exhibits a non-ideal shape with statistical fluctuations and a slower decay component, while the discriminator maintains a consistent triggering behavior when the threshold is exceeded.

The behaviour of the discriminator with a test pulse is shown in Figure 4. A similar response is observed when processing a real scintillation signal acquired with a SiPM, as shown in Figure 5.

The similarity between Figure 4 and Figure 5 refers to the discriminator behavior rather than to the detailed analog waveform shape: in both cases, the discriminator generates a logic output when the analog signal at Ana_out exceeds the selected threshold, with a comparable triggering sequence and timing relation between the analog pulse leading edge and the logic transition. The injected test pulse provides a controlled reference input, whereas the real SiPM signal exhibits the expected statistical fluctuations and a slower tail associated with the detector response.

### 3.3. Peak Hold

To overcome the intrinsic limitations of microcontroller ADC sampling rates relative to the SiPM signal duration, NanoArduSiPM employs a peak-hold circuit. This analog block stretches the short SiPM pulse to a duration on the order of microseconds, allowing its amplitude to be reliably sampled by the SoC 12-bit ADC operating at 2 MSPS. A programmable reset mechanism enables adaptation of the signal shaping to the scintillator and application-specific requirements. Under the nominal configuration used in this work, the peak-hold stage fixes the signal amplitude for approximately 2 µs [22], resulting in a system dead time of the same order. To ensure deterministic operation, a programmable register is implemented to control the reset interval of the peak-hold stage. This compensates for small variations in the effective acquisition time due to system operating conditions and allows the dead time of the amplitude acquisition branch to be set to a fixed and programmable value, typically in the range of 2–5 µs. It is worth noting that this dead time affects only the amplitude acquisition branch, as it is introduced by the peak-hold and ADC conversion process. In contrast, the timing branch based on the fast discriminator remains continuously active and is not limited by this effect, allowing uninterrupted event counting and timing measurements. The peak-hold stage is implemented as a discrete analog circuit, in which the output amplitude and temporal response are governed by the charge storage and discharge dynamics of the holding capacitor and the associated resistive network. The effective gain is therefore determined by the closed-loop configuration of the active stage and by the values of the surrounding passive components, which define both the amplitude scaling and the integration and reset time constants. In the current implementation, the circuit is designed to provide an effective gain close to unity, ensuring a direct mapping of the input pulse amplitude to the ADC input range. As a result, the transfer characteristic is intrinsically fixed by the analog front-end design, while maintaining proper matching between the SiPM signal and the ADC dynamic range.

**Figure 6:**
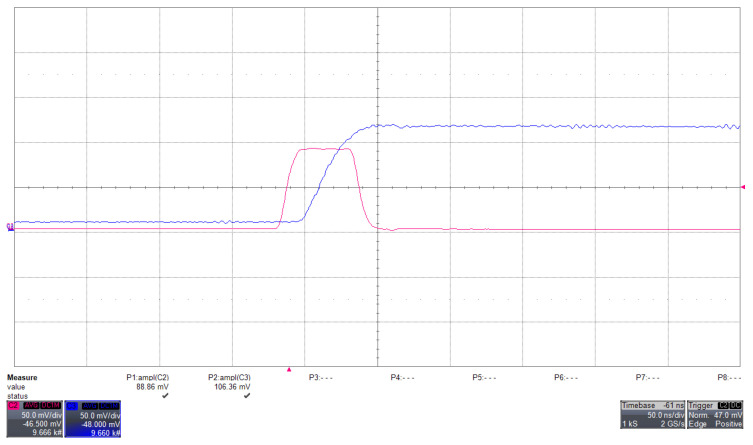
(Colour online) Peak-hold response to a test pulse injected at the LNA input, with the SiPM high voltage disabled. The fuchsia trace shows the analog signal at the LNA output (Ana_out_), while the blue trace represents the peak-hold output. Both signals are displayed with a vertical scale of 50 mV/div and a horizontal scale of 50 ns/div, as measured with a Lecroy WaveRunner HRO 64 Zi oscilloscope (1 GHz bandwidth). The peak-hold circuit stretches the fast input pulse, maintaining its maximum amplitude over an extended time interval (from the nanosecond to the microsecond scale), thus enabling reliable sampling by the ADC. The output amplitude is slightly increased with respect to the input signal, corresponding to a gain of approximately 1.2.

The peak-hold behavior is shown in Figure 6, where the output waveform tracks the pulse maximum and maintains the amplitude long enough for ADC acquisition.

## 4. Firmware and Digital Signal Processing

A key strength of the ArduSiPM architecture lies in its firmware-driven approach, where system functionality and complexity are largely implemented at the software level rather than in dedicated hardware. This design paradigm enables a high degree of flexibility and adaptability, allowing the same hardware platform to be reconfigured for different applications through custom firmware implementations. In particular, advanced functionalities such as event selection, coincidence and veto logic, real-time data analysis, and on-board histogramming are implemented directly within the embedded processing layer. In this framework, the firmware is not limited to data acquisition control, but plays a central role in defining the behavior of the detector, effectively transforming the device into a configurable and scalable processing node within distributed detection systems.

### 4.1. Firmware Architecture and Role

The firmware represents the digital processing layer of the NanoArduSiPM system, complementing the analog front-end and enabling fully embedded data acquisition and analysis. In the overall three-layer architecture, consisting of the detection stage, the discrete analog front-end, and the System-on-Chip (SoC), the firmware operates within the SoC to control data flow, manage acquisition, and perform real-time processing of detector signals. Unlike conventional SiPM readout systems, where data acquisition and processing are typically performed by external electronics, the NanoArduSiPM firmware implements these functionalities locally. This approach enables edge computing capabilities, reducing the need for external data acquisition systems and minimizing data transfer requirements. As a result, the device operates as a self-contained detection and processing unit, particularly suitable for resource-constrained environments such as space-based or distributed applications. A key feature of the ArduSiPM architecture is the ability to implement system functionality at firmware level rather than through dedicated hardware. This design paradigm provides a high degree of flexibility, allowing the same hardware platform to be reconfigured for different experimental conditions and applications through custom firmware implementations. In particular, advanced functionalities such as event selection, coincidence and veto logic, real-time histogramming, and data reduction are implemented within the firmware, enabling a configurable and adaptable detection system. In this framework, the firmware is not limited to low-level control tasks, but plays a central role in defining the behavior of the detector. By integrating acquisition, processing, and decision-making within the same embedded platform, NanoArduSiPM can operate as an autonomous processing node, supporting scalable and distributed detection architectures based on multiple coordinated units.

### 4.2. Event Acquisition and Data Flow

The firmware manages the acquisition of detector signals by coordinating the interaction between the analog front-end and the internal peripherals of the SoC. An event is defined as the detection of a SiPM pulse exceeding a programmable threshold set by the SoC DAC. The fast discriminator stage generates a digital trigger, which is processed by the SoC for event counting and rate measurements, with architectural support for time-tagging. Once an event is triggered, the signal is processed along two parallel paths. In one branch, the discriminator output is processed by the internal counters of the microcontroller for event counting and rate measurements, with support for time-tagged acquisition at the architectural level.

In the amplitude branch, the analog signal, stretched by the peak-hold circuit to match the temporal characteristics of the ADC, is digitized by the internal analog-to-digital converter. The resulting digital value represents the pulse amplitude associated with the detected event. This coordinated handling of multiple data streams is performed in real time, ensuring continuous acquisition without the need for external control systems. This integrated acquisition chain enables embedded pulse analysis in terms of amplitude and rate within a compact and self-contained architecture.

### 4.3. Online Histogramming with Event Selection

Although the system allows the characteristics of each individual event to be recorded, data volume considerations make on-board data reduction essential in many applications. In this context, a firmware-based histogramming approach provides an efficient and flexible solution. In the following, the term spectrum.

### 4.4. Communication Interfaces

NanoArduSiPM inherits the full suite of communication interfaces available on the SAMV71 SoC. High-speed USB 2.0 is used for primary data transfer and configuration, supporting rates up to 480 Mbps. In addition, UART, I^2^C and CAN interfaces are provided for system integration in embedded applications. In the satellite configuration, a Serial Peripheral Interface (SPI) has been selected as the primary communication link between the detector and the satellite On-Board Computer (OBC), providing a deterministic and fully synchronous data exchange. This interface ensures deterministic operation with well-defined and predictable latency, and its master–slave architecture naturally aligns with the hierarchical organization of on-board data handling systems.

Such characteristics make SPI particularly suitable for payload subsystems requiring event-driven acquisition and deterministic synchronization with spacecraft reference clock signals, where asynchronous protocols such as UART may introduce variable delays and jitter.

Conversely, the USB and wireless interfaces are primarily intended for ground-based use, supporting laboratory testing, calibration and stand-alone operation of NanoArduSiPM as a scientific or diagnostic instrument. Wi-Fi, Bluetooth, and LoRa connectivity are implemented as easily attachable add-on modules, providing versatile wireless access for configuration, data acquisition and distributed sensor networks during ground operations or field deployments. This dual-interface architecture enables a seamless transition from laboratory development to in-orbit deployment, minimizing hardware modifications while maintaining full access to configuration and diagnostic capabilities throughout all project phases. Altogether, these interfaces provide comprehensive configurability, operational flexibility and seamless integration in both terrestrial and space applications.

## 5. Assessing NanoArduSiPM: Characterization and Performances

The performance of NanoArduSiPM was thoroughly evaluated through a dedicated characterization [22]. This assessment aimed to verify improvements over previous versions while ensuring the device suitability for its target applications. Key aspects of the evaluation included the performance of the analog front-end and the characterization of SiPM signal amplitudes. Although the timing branch is physically implemented, this work focuses on the validation of the amplitude and rate processing chains.

### 5.1. Front-End Stage Linearity and Amplification

Before analyzing the full detector response including the SiPM, it is essential to characterize the electronic signal-processing chain under controlled conditions.

In this work, this is achieved by injecting electrical test pulses at the front-end input, which reproduce the equivalent voltage transient present at the SiPM readout node after current-to-voltage conversion.

This approach allows the intrinsic behavior of the analog front-end and the digitization chain to be evaluated independently of the stochastic nature of the SiPM signals, ensuring a well-defined and reproducible input condition for the system-level characterization.

To this end, fast electrical pulses, designed to reproduce the temporal characteristics of typical SiPM signals while providing fully controlled and reproducible input conditions, were injected at the input of the front-end. The response of the system was then analyzed in terms of amplitude linearity and gain. To evaluate the analog signal path preceding the digitization stage of the SAMV71 ADC, the behavior of the key front-end components, namely the low-noise amplifier and the peak-hold circuit, was characterized using controlled electrical test pulses injected at the input of the amplification stage, with the SiPM bias disabled.

In this context, the input voltage $V_{in}$ refers to the amplitude of the injected test signal, which reproduces the equivalent voltage developed at the SiPM readout node after current-to-voltage conversion.

The gain *m* is defined as the ratio between the output voltage $V_{out}$, measured at the output of the considered stage, and the input voltage $V_{in}$:

(1) $$m=\frac{V_{out}}{V_{in}}$$

In this definition, *m* refers to the voltage gain of the specific stage under consideration, and is evaluated independently for each block of the analog front-end.

It is important to note that this definition refers to the response of the analog front-end to injected electrical signals and does not directly represent the intrinsic response of the SiPM, which produces current pulses. Rather, it provides a characterization of the voltage-domain behavior of the readout chain under controlled conditions.

**Figure 7:**
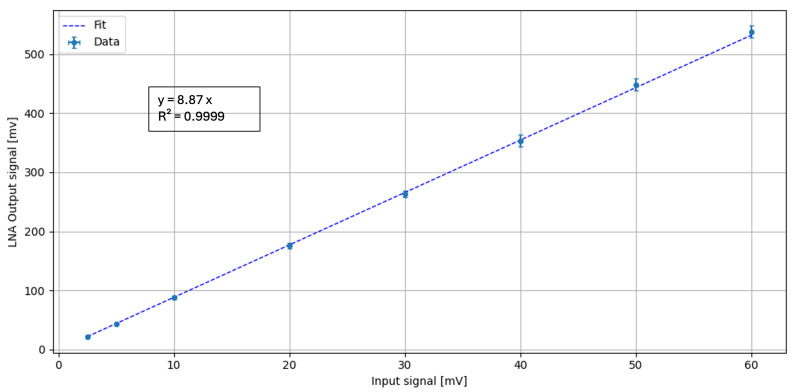
Linear regression of LNA output as a function of the signal input amplitude. A fast 40 ns signal pulse, is used as input to simulate a typical SiPM pulse.

Fast electrical pulses of 40 ns width, representative of typical SiPM output signals, were injected with varying amplitudes to assess the linearity of the front-end response. As shown in Figure 7, the output of the LNA displays a linear dependence on the input signal amplitude. The regression fit yields a slope of m=8.87±0.09, which corresponds to the amplification gain of the LNAn, with a correlation coefficient R=0.9999, indicating excellent linearity.

**Figure 8:**
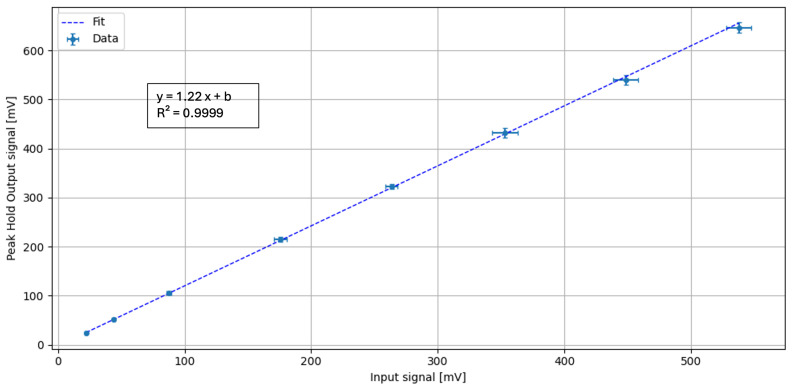
Linear regression of peak hold output as a function of signal input amplitude. A fast 40 ns signal pulse, is used as input to simulate a typical SiPM pulse. The offset is a feature of the peak hold circuit that depends on each individual device and can be compensated.

The subsequent peak-hold stage, illustrated in Figure 8, also demonstrates a linear response with R=0.9999, and a fitted slope of m=1.22±0.01, corresponding to the effective voltage gain of the peak-hold stage under the same test conditions.

The observed baseline offsets can be compensated using the internal 10-bit DAC provided by the SAMV71, thereby restoring the baseline and improving the system’s effective dynamic range.

### 5.2. ADC Calibration

The analog signal processing chain of NanoArduSiPM builds upon the architectural concept introduced over a decade ago in the first ArduSiPM developments. This design philosophy has proven remarkably forward-looking, anticipating the steady advances in mixed-signal integration and component miniaturization that now enable compact and efficient implementations. Compared to the ADCs integrated in earlier SoCs, modern converters offer significantly improved performance and calibration capabilities, with higher sampling rates, lower noise, enhanced linearity and advanced self-calibration. These advances have provided a major boost to the overall system performance and flexibility of the analog front-end. The SoC integrates two analog input paths to the ADC, each equipped with an independent Sample-and-Hold circuit, a 10-bit programmable DAC, and a Programmable Gain Amplifier (PGA), selectable via an internal multiplexer. For the scope of this characterization, only one analog channel is employed. Offset compensation is achieved by adjusting the internal DAC to align the ADC baseline to zero. This calibration ensures that a 0 mV input corresponds to 0 ADC code, independent of the selected PGA gain. The ADC provides 12-bit resolution (4096 channels) over a 3.3 V range, while the sampling rate is 2 MS/s, allowing accurate digitization of signals.

To characterize the digitization response of the NanoArduSiPM system, a controlled electrical test signal was injected at the input of the analog front-end (i.e., at the input of the low-noise amplifier), following the same configuration described in Section 5.1. The signal propagates through the low-noise amplifier and the peak-hold stage before being digitized by the internal ADC of the SAMV71.

In this context, the term “overall amplification chain” refers to the complete signal path from the LNA input node to the digital output of the ADC, including the analog front-end stages and the ADC conversion.

A 1 kHz pulse with 100 ns width was applied to the input, and its amplitude was varied over the operating range. For each amplitude step, the corresponding ADC output value was recorded in digital counts.

A linear regression was then performed on the input–output characteristic to evaluate the linearity of the system response.

This characterization does not separate the contributions of the analog front-end and the ADC, but instead provides a system-level evaluation of the complete acquisition chain as implemented in the NanoArduSiPM architecture.

**Figure 9:**
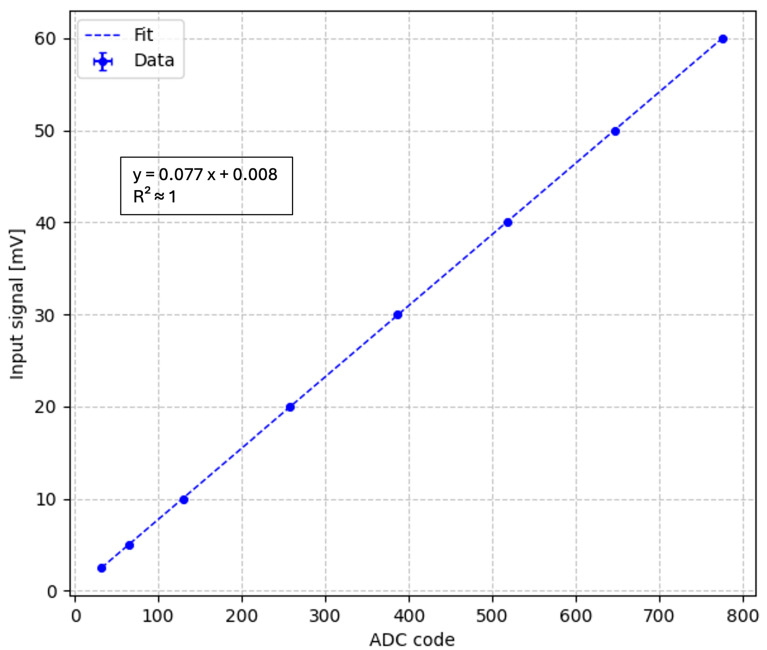
Linear regression of ADC digital output as a function of the input signal amplitude. The error bars are smaller than the marker size and are therefore not visible in the plot.

In the case of PGA = 1, the DAC offset was set to 519 codes. The response is linear up to 650 mV, and the slope of the linear fit corresponds to an effective conversion factor of 0.077 mV/code, with a negligible intercept, as shown in Figure 9. These measurements confirm the linearity of the overall acquisition chain and quantify the effective conversion characteristic from input signal amplitude to ADC output, ensuring reliable digitization within the operating range of NanoArduSiPM.

## 6. System-Level Characterization and Correction of ADC Non-Linearity

The NanoArduSiPM acquisition chain exhibited a highly stable and reproducible behavior during testing; however, a detailed system-level characterization revealed the presence of intrinsic localized ADC non-uniformities.

This section highlights a system-level issue that becomes relevant in highly integrated, compact detection architectures: intrinsic ADC non-uniformities can generate localized spectral distortions when the converter output is directly histogrammed for spectroscopic reconstruction. In NanoArduSiPM, this effect is corrected through a firmware-based strategy that requires no modification of the underlying hardware architecture.

However, a detailed system-level characterization revealed the presence of intrinsic localized ADC code non-uniformities consistent with differential non-linearity (DNL), appearing as under-populated bins. These appeared as systematic deficits in the number of recorded events under constant and uniform input conditions, indicating non-uniformities in ADC behavior. While such effects may have little impact on typical monitoring or counting applications, in nuclear spectroscopy even a small inefficiency on a single ADC channel translates into an unidentified gap in the reconstructed energy spectrum, potentially affecting spectral continuity and resolution.

### 6.1. Experimental Setup for the Characterization of ADC Channel Non-Uniformity

**Figure 10:**
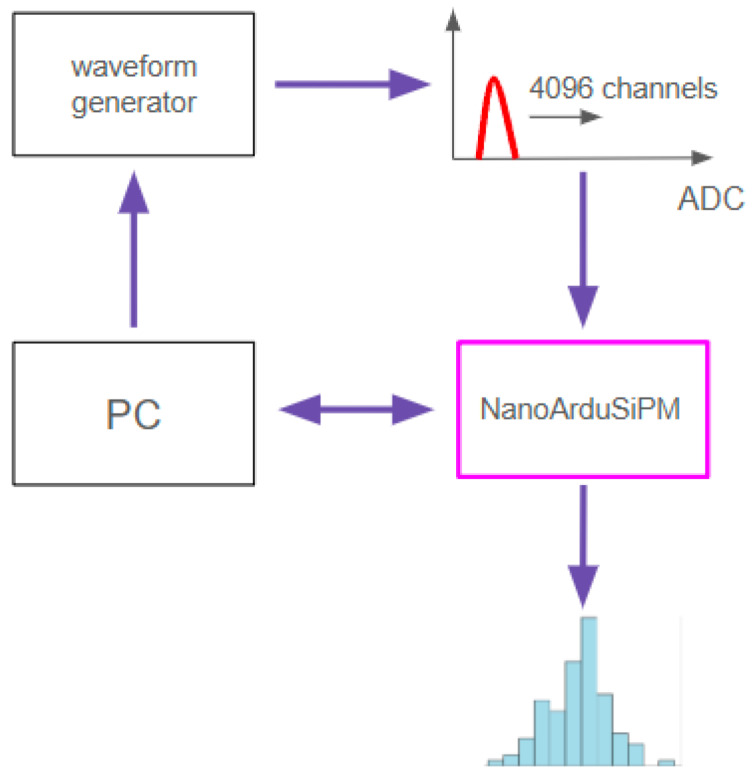
Block diagram of the automated setup used to characterize ADC channel efficiency. The waveform generator produces rectangular pulses of controlled amplitude and frequency, acquired through the ADC and analyzed by the NanoArduSiPM.

To investigate this effect, an automated measurement procedure was implemented using rectangular input pulses of fixed width and frequency, with gradually increasing amplitude. The experimental setup used to characterize the ADC channel inefficiencies is schematically shown in Figure 10. It includes a PC-controlled waveform generator, the ADC acquisition chain and the NanoArduSiPM for data processing and histogram generation.

### 6.2. Origin of the Effect

The observed behavior is consistent with intrinsic limitations of the ADC architecture, specifically related to linearity errors induced by internal parasitic capacitances. This interpretation is supported by direct feedback from the Microchip ADC design team, confirming that the effect is inherent to the converter.

According to the manufacturer’s analysis, the maximum integral non-linearity occurs in two regions located at approximately one-eighth and seven-eighths of the full-scale range. These regions are also expected to exhibit increased differential non-linearity (DNL), which can lead to local missing or under-populated codes.

**Figure 11:**
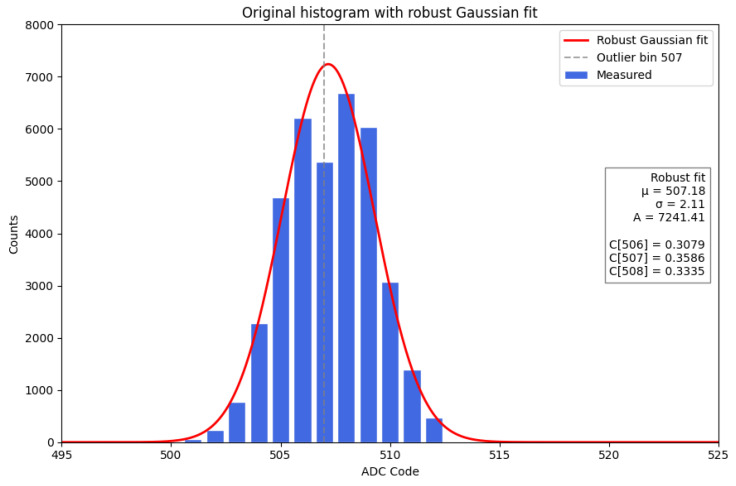
Raw ADC histogram showing non-uniform bin population due to differential non-linearity (DNL). A clear deficit is visible at ADC channel 507, indicating an inefficient channel and producing a localized gap in the spectrum.

The observed behavior is consistent with differential non-linearity (DNL) effects, occurring in regions where the integral non-linearity (INL) is known to peak, in agreement with the anomalies observed experimentally. The affected regions were identified around ADC codes 512 and 3584 (0x202 and 0xE00), with device-to-device variations of about ±10 codes. Since this effect is intrinsic to the ADC architecture and cannot be mitigated at the hardware level in the current system, a firmware-based correction approach is adopted. The consistency between the measured spectra and the manufacturer’s characterization demonstrates that the inefficiency is not related to firmware or external circuitry, but is instead an intrinsic feature of the ADC design. When the converter output is directly used for spectroscopic reconstruction, these localized non-uniformities manifest as artificial gaps or distortions in the energy spectrum. A clear experimental example of this effect is provided by a specific chip mounted on a Nano ArduSiPM board, where a pronounced inefficiency is observed at channel 507 (see Figure 11).

### 6.3. Firmware-Based Correction of ADC Non-Uniformity

To compensate for the localized ADC inefficiencies described above, a computationally efficient local correction algorithm suitable for real-time firmware implementation is applied at the histogram readout stage. These non-uniformities, which manifest as distortions in reconstructed energy spectra, are addressed by applying a localized redistribution of counts. The correction operates by transferring a fraction of events from inefficient channels to their immediate neighbors, based on predefined redistribution weights. These weights are derived from a local model of the expected distribution, as described in the following section. The algorithm is designed to restore local count density while preserving the global integral of the spectrum. To minimize any impact on spectral resolution, the correction is strictly confined to the immediate vicinity of the affected channels.

Importantly, this procedure is applied exclusively during the readout phase. The raw acquisition data and the analog processing chain remain completely unaltered, ensuring that the correction is fully reversible and does not interfere with the underlying measurement.

A consistency check was performed by comparing the total number of events recorded in the histogram with the counts obtained from the discriminator-based counter, showing full agreement within statistical uncertainties. This confirms that the amplitude acquisition chain preserves event integrity and does not introduce losses.

### 6.4. Robust Outlier Correction and Bin Redistribution

Anomalous bins in the ADC histogram are corrected through a two-step procedure: estimation of the expected distribution, followed by local redistribution of counts under integral conservation.

The reference shape is modeled by a Gaussian function $f(x_{i})$, as expected when sampling a fixed-amplitude input during the amplitude scan, where the observed distribution is dominated by electronic noise and ADC response fluctuations. The Gaussian parameters are determined through an iterative robust reweighting procedure, where Cauchy-type weights are updated from the normalized residuals at each step until convergence, reducing the impact of bins affected by ADC inefficiencies.

The correction is applied locally to the neighborhood of an anomalous bin *k*. The correction is restricted to a three-bin window (k−1,k,k+1), which represents the minimal local neighborhood capturing the ADC-induced distortion, while preserving spectral resolution by avoiding unnecessary broadening of the distribution.

The total counts in the 3-bin window are:

(2) $$N_{k}^{3\mathrm{bin}}=\sum_{i=k-1}^{k+1}n_{i}$$

Redistribution coefficients are derived from the fitted model:

(3) $$C_{i}=\frac{f(x_{i})}{\sum_{m=k-1}^{k+1}f\left(x_{m}\right)},\;i\in \left\{k-1,k,k+1\right\}$$

The corrected bin contents are then obtained by redistributing the total counts:

(4) $$n_{i}^{\prime }=N_{k}^{3\mathrm{bin}}\cdot C_{i}$$

which explicitly enforces local integral conservation:

(5) $$\sum_{i=k-1}^{k+1}n_{i}^{\prime }=N_{k}^{3\mathrm{bin}}=\sum_{i=k-1}^{k+1}n_{i}$$

For firmware implementation, the redistribution is performed directly by assigning the total counts in the local window to each bin according to the precomputed coefficients:

(6) $$n_{k-1}^{\prime }=C_{k-1}\cdot N_{k}^{3\mathrm{bin}},\;n_{k}^{\prime }=C_{k}\cdot N_{k}^{3\mathrm{bin}},\;n_{k+1}^{\prime }=C_{k+1}\cdot N_{k}^{3\mathrm{bin}}$$

with

(7) $$C_{k-1}+C_{k}+C_{k+1}=1$$

The identification of inefficient channels and the corresponding coefficients are determined through a device-specific calibration and stored in the microcontroller flash memory for use during runtime.

This formulation is computationally efficient and exactly equivalent to the model-based redistribution.

**Figure 12:**
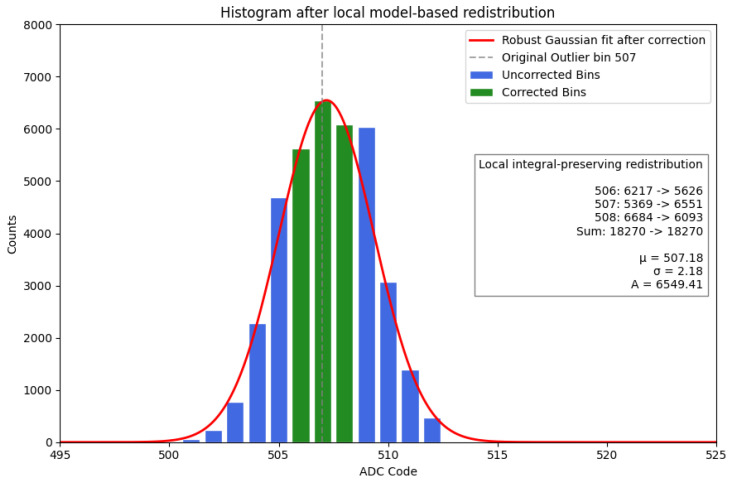
ADC histogram after local integral-preserving (3-bin) correction. Counts from adjacent channels are partially redistributed according to the normalized coefficients $C_{i}$ within the local 3-bin window (k−1, *k*, k+1). This restores spectral continuity and improves agreement with the expected distribution.

The result of the correction is shown in Figure 12, where the discontinuity is removed and the distribution recovers its expected smooth behavior. To quantify the magnitude of the observed local non-uniformity, an effective differential non-linearity can be estimated from the deficit of the anomalous bin with respect to the fitted expected distribution. Denoting by $n_{k}$ the measured counts in bin *k* and by $f(x_{k})$ the corresponding value of the fitted local model (used to derive the coefficients $C_{i}$ in Equation (3)), the effective DNL can be approximated as:

(8) $$DNL_{k}^{\mathrm{eff}}\approx \frac{n_{k}}{f(x_{k})}-1$$

Equivalently, using the local three-bin normalization defined in Equations (2) and (3), one can write:

(9) $$DNL_{k}^{\mathrm{eff}}\approx \frac{n_{k}}{N_{3\mathrm{bin}}^{\mathrm{obs}}\;C_{k}}-1$$

For the anomalous channel visible in Figure 11 and corrected in Figure 12 (ADC code 507), this yields

(10) $$DNL_{507}^{\mathrm{eff}}\approx -0.18,$$

corresponding to an effective bin width of approximately 0.82LSB.

It is important to note that the correction affects only the histogram representation. To preserve full information, the raw counts of the corrected region are appended to the output data stream at the end of the histogram transfer. This guarantees full traceability between corrected and original data and allows for offline validation or alternative correction strategies.

The output histogram is transmitted using zero suppression, ensuring efficient data transfer without affecting the correction procedure.

### 6.5. SiPM Signal Amplitude

Following the calibration of the threshold DAC and the Analog Front End ADC, the analysis of the signal amplitude was undertaken to correlate the number of detected photoelectrons with the measured signals. The threshold DAC, characterized by its fine resolution, serves as a precise instrument to link the signal amplitude to the corresponding number of photoelectrons fired. Given that the SiPM signal amplitude is quantized as an integer multiple of the single-cell response, lower amplitude signals, representing fewer simultaneous avalanches, are statistically more frequent.

A threshold scan was performed to investigate the count rate as a function of the discriminator level. Increasing the threshold progressively excludes lower-amplitude signals, effectively discriminating against smaller photoelectron coincidences. This process results in a characteristic stepwise count rate profile where each discrete drop corresponds to a specific number of photoelectrons.

**Figure 13:**
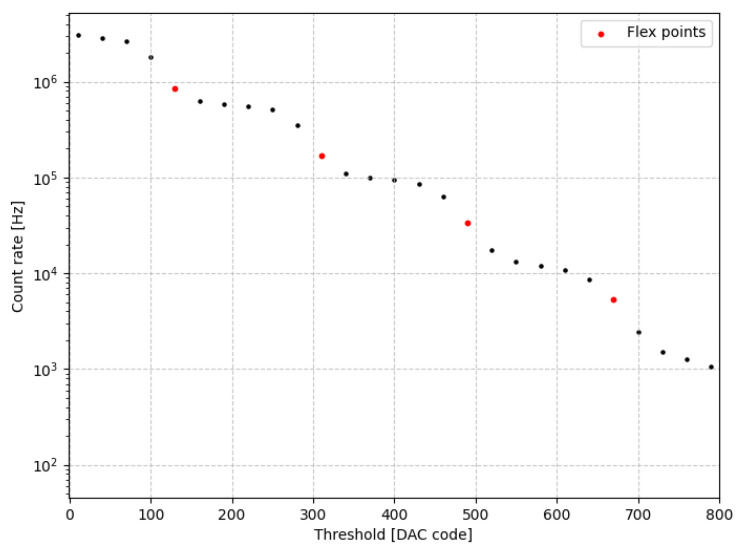
Flexpoints at DAC codes 130, 310, 490, 670.

The SiPM used for this setup was the S13360-1350PE (Hamamatsu Photonics K.K., Hamamatsu, Japan) [23]. In addition, to extend the dynamic range and resolve higher coincidence numbers, the experimental setup was enhanced by introducing pulsed LED illumination, controlled by a PDL 800-B (PicoQuant GmbH, Berlin, Germany) [24]. The resulting threshold scan is shown in Figure 13, reveals the first four coincidence peaks clearly. The stepwise structure is consistent with the analytical model of multiple-coincidence dark noise in SiPMs presented in [25], which predicts the relative positions and amplitudes of the coincidence peaks observed in threshold scans.

By identifying the flex points in the threshold scan curve and converting these DAC code differences to voltage, the amplitude of a single photoelectron peak was determined to be

$$\Delta_{\mathrm{peak}}=A_{1\;p.e.}=2.7\pm 0.2\;\mathrm{mV},$$

with the first peak located at approximately 2.8±0.2mV.

**Figure 14:**
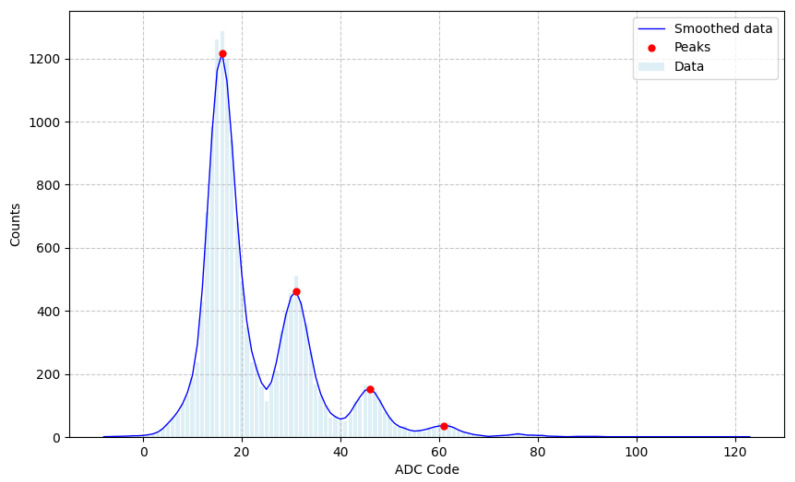
Smoothed ADC spectrum with peaks at codes 16, 31, 46, 61.

Furthermore the internal ADC of the NanoArduSiPM plays a crucial role in local data processing and quantification of photon counts. To cross-validate the threshold scan results, we also analyzed the spectrum obtained by digitizing the SiPM signal after amplification stages. Illuminated with low-intensity LED pulses under the same conditions, the ADC spectrum in Figure 14 exhibits up to the fourth coincidence peak. Applying a one-dimensional Gaussian filter to smooth the data and locate the peaks and converting the ADC codes to voltage, it yields a single-photoelectron amplitude of

$$\Delta_{\mathrm{peak}}=A_{1\;p.e.}=2.6\pm 0.3\;\mathrm{mV},$$

with the first peak at 2.7±0.3mV.

The excellent agreement between the peak amplitudes measured via the threshold DAC scan and the ADC spectrum demonstrates the validity of the NanoArduSiPM’s signal processing customized chain in resolving single photoelectrons. This analysis also confirms that NanoArduSiPM can reliably discriminate single and multiple photoelectron events, an essential feature for applications requiring precise photon counting and signal characterization.

The main quantitative performance parameters of the NanoArduSiPM are summarized in Table 1.

### 6.6. Preliminary Scintillation Measurements

To demonstrate the operation of NanoArduSiPM as a complete scintillation-based detector system, preliminary measurements were performed using a cylindrical LaBr_3_(Ce) scintillator with 25 mm diameter and 25 mm height, coupled to a Hamamatsu S13360-6025CS SiPM and directly read out by the NanoArduSiPM acquisition chain.

**Figure 15:**
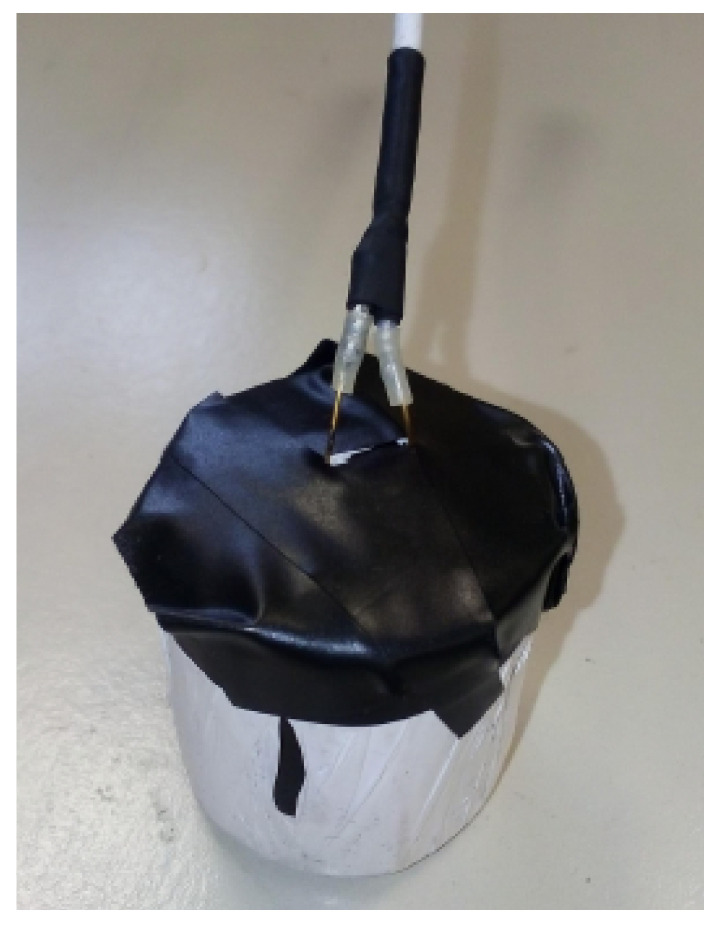
LaBr_3_(Ce) scintillator coupled to a Hamamatsu S13360-6025CS SiPM used for the preliminary measurements.

Figure 15 shows the detector assembly, including the LaBr_3_(Ce) scintillator coupled to the SiPM. The compact configuration demonstrates the suitability of the system for lightweight and space-constrained applications requiring fully integrated scintillation detection.

**Figure 16:**
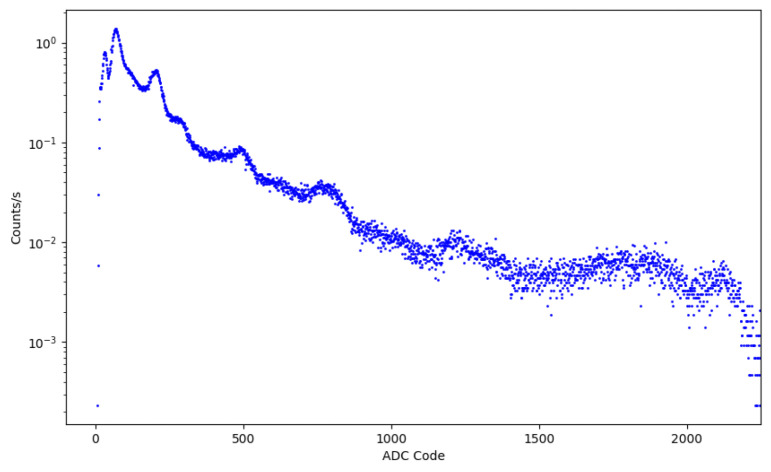
^232^Th spectrum acquired with NanoArduSiPM coupled to a LaBr_3_(Ce) scintillation detector after background subtraction.

A preliminary spectrum of a ^232^Th radioactive source is reported in Figure 16. The ^232^Th source was selected because of its rich gamma emission structure extending over a broad energy range, making it suitable for a preliminary qualitative evaluation of the detector response. Several characteristic structures consistent with the expected ^232^Th spectrum are visible over a broad energy range, confirming the capability of the system to acquire and process real scintillation signals in standalone operation.

Although the primary scope of the present work is the validation of the compact integrated architecture and embedded acquisition chain rather than a complete spectroscopic characterization, these measurements provide an initial experimental validation of the detector performance under realistic operating conditions.

A more detailed spectroscopic characterization of the NanoArduSiPM system coupled to inorganic scintillators will be presented in a dedicated future publication.

## 7. Conclusions

NanoArduSiPM demonstrates that a fully integrated scintillation-based detector, including signal conditioning, digitization, and embedded processing, can be implemented within a compact 42×36×3 mm, 7 g module while preserving high performance in terms of signal linearity and photon-counting capabilities. By combining a discrete analog front-end with a high-speed SoC, the system provides complete on-board acquisition, processing, and communication capabilities in a form factor suitable for embedded and space-constrained applications.

The characterization results confirm the performance of the analog signal chain, which exhibits excellent linearity and enables accurate signal amplification and digitization. The peak-hold stage adapts the nanosecond-scale SiPM signals to the ADC timescale, allowing reliable amplitude measurements with the embedded converter. The system is able to resolve single-photoelectron signals both through threshold scans and direct ADC analysis, confirming its capability for single-photon detection.

Although based on the established ArduSiPM concept, this work goes beyond a simple incremental improvement. The achieved level of miniaturization, combined with fully embedded acquisition, processing, and event-level decision capabilities, enables a different operational paradigm. In this regime, each module operates as an autonomous and programmable detection node, rather than as a passive readout channel within a centralized acquisition system.

The reduced size, low power consumption, and firmware-driven architecture allow NanoArduSiPM to operate as an autonomous processing node. This enables local data processing, event selection, and histogramming without the need for external acquisition systems, supporting applications ranging from laboratory measurements to distributed sensing and satellite payloads.

While the system demonstrates characteristics compatible with compact space platforms, its radiation tolerance has not been experimentally assessed in this work and will be addressed in future developments.

More generally, this work shows that tightly integrated analog front-end design and embedded processing can be co-designed to realize compact and self-contained detection units. These units can be replicated and interconnected to form scalable and distributed measurement systems, where system functionality is largely implemented at firmware level rather than through dedicated hardware.

Future work will address the experimental validation of multi-module synchronization and detailed timing performance, which, although supported at the architectural level, are not yet quantitatively characterized. These aspects are currently under investigation and will further extend the capabilities of the proposed architecture.

## Figures and Tables

**Table 1:** Summary of the main quantitative performance parameters of NanoArduSiPM, including intrinsic system characteristics and representative benchmark measurements obtained with a Hamamatsu S13360-1350PE SiPM.

Parameter	Value
Dimensions	42×36×3 mm
Mass	7 g
Typical power consumption (nominal conditions)	∼300 mW
LNA gain	8.87±0.09
Peak-hold gain	1.22±0.01
Analog front-end linearity (*R*)	0.9999 (LNA), 0.9999 (peak-hold)
ADC resolution	12 bit (4096 channels)
ADC sampling rate	2 MS/s
*Benchmark measurements (Hamamatsu S13360-1350PE SiPM)*	
Single-photoelectron threshold step (DAC)	∼180 counts
Single-photoelectron peak spacing (ADC, PGA ×4)	∼16 counts

## Data Availability

The data presented in this study are available on request from the corresponding author.
